# Editorial: Genetic engineering, pretreatment, thermochemical, and biochemconversion for lignocellulose valorization

**DOI:** 10.3389/fbioe.2023.1265271

**Published:** 2023-08-21

**Authors:** Xiaojun Shen, Jia-Long Wen, Chen Huang, Arthur J. Ragauskas, Chaofeng Zhang

**Affiliations:** ^1^ Beijing Key Laboratory of Lignocellulosic Chemistry, Beijing Forestry University, Beijing, China; ^2^ Jiangsu Province Key Laboratory of Biomass Energy and Materials, Institute of Chemical Industry of Forest Products, Chinese Academy of Forestry, Nanjing, China; ^3^ Department of Chemical and Biomolecular Engineering, University of Tennessee Knoxville, Knoxville, TN, United States; ^4^ Department of Forestry Wildlife and Fisheries, Center for Renewable Carbon, The University of Tennessee Institute of Agriculture, Knoxville, TN, United States; ^5^ Joint Institute for Biological Science, Biosciences Division, Oak Ridge National Laboratory, Oak Ridge, TN, United States; ^6^ Jiangsu Co-Innovation Center of Efficient Processing and Utilization of Forest Resources, College of Light Industry and Food Engineering, Nanjing Forestry University, Nanjing, China

**Keywords:** lignocellulose, fractionation, structural characterization, genetic engineering, enzymatic hydrolysis, thermconversion, bioconversion

## Introduction

Lignocellulosic biomass, comprising cellulose, hemicelluloses, and lignin, is a remarkable renewable resource with the potential to replace fossil feedstocks and drive a sustainable bioeconomy (Queneau and Han, 2022; Shen et al., 2022). However, the complex structure of lignocellulose presents challenges in effectively converting it into high-value products (Huang et al., 2022; Cai et al., 2023). The need to unlock its full potential has led to the development of biorefineries, aiming to fractionate lignocellulose and convert it into bioenergy, biomaterials, and biochemicals. Since the complex compositional structure of lignocellulosic biomass and biomass recalcitrance severely inhibited their effective conversion and selective production of high-value products. To overcome this disadvantage, using genetic and chemical approaches can modify cell wall composition and change interactions between the major cell wall polymers-cellulose, hemicelluloses and lignin, which would reduce the biomass recalcitrance and facilitate the subsequent fractionation and conversion of the feedstocks (Huang et al., 2022). Despite its vast potential, only a small fraction of lignocellulose is currently valorized, with the majority being wasted or burned. To address this issue, advanced technologies, including genetic, biotechnological, and chemical approaches, are required to fractionate and convert lignocellulose into valuable products efficiently.

The Research Topic “*Genetic engineering, pretreatment, thermochemical, and biochemconversion for lignocellulose valorization*” covers the new biotechnological approaches for value-added utilization of biomass, new biomass fractionation techniques for improving lignin and cellulose quality, new catalytic biomass valorization strategies, and applications of lignocellulosic materials and derivatives in related fields. Here, we sincerely appreciate the 61 authors for their excellent work on this Research Topic. Following are the highlights drawn from their contributions to this Research Topic.

## New biotechnological approaches for value-added utilization of biomass

In the enzymatic hydrolysis process of cellulose, the presence of lignin leads to non-specific adsorption of cellulose hydrolyzing enzymes, resulting in low cellulose conversion rates. Additionally, water-soluble lignin in the liquid phase also acts as an inhibitor of enzymatic hydrolysis. Therefore, the existence of lignin has long been a hindrance to the conversion of carbohydrate biomass. Tang et al. addressed this issue by subjecting pretreated residues to enzymatic hydrolysis for glucose production, during which lytic polysaccharide monooxygenase (LPMO) was introduced. Despite the presence of lignin, the cellulose enzymatic hydrolysis conversion rate was increased by 25%, surpassing the enhancement achieved with microcrystalline cellulose by 14%. This significant improvement is mainly attributed to lignin and its derivatives serving as electron donors, facilitating LPMO in catalyzing the oxidative cleavage of glycosidic bonds in cellulose, thus generating more targets for hydrolysis. This study offers a novel approach to overcoming the challenges posed by lignin during carbohydrate enzymatic conversion processes and provides new insights into the utilization of lignin in such contexts. Besides, Reger et al. developed a novel hybrid process strategy that tackles the key challenges of current biomanufacturing of either low productivity or high media consumption, representing a new and innovative approach for future process intensification efforts. In addition, most enzymes used for carbohydrate hydrolysis are mesophilic enzymes, limiting their industrial applications. Zhang et al. utilized thermophilic filamentous fungi to produce xylanase and obtained a heat-tolerant mutant M 2103, through atmospheric and room temperature plasma (ARTP) mutagenesis. The M 2103 mutant exhibited stable enzymatic activity for xylanase within a temperature range of 70°C–85°C, with an optimum temperature of 75°C, surpassing the wild-type strain by 15°C. The xylanase activity of the mutant increased by 21.71% compared to the original strain. This provides an alternative biocatalyst for producing xylo-oligosaccharides and holds promising potential in the mutagenesis of thermostable mutants. In addition to agricultural and forestry waste, cigar filler leaves, when subjected to fermentation using nine different aroma-producing yeasts, were found to possess distinct profiles that can enhance the quality of cigar filler leaves Yao et al.

## New biomass fractionation techniques for improving lignin and cellulose quality

In plant cell walls, hemicellulose and lignin are linked by covalent and hydrogen bonds to form a heterogeneous structure, which wraps around cellulose, creating a robust “natural recalcitrance barrier.” This barrier significantly limits the efficiency of biomass refinement. Pretreatment is essential to overcome biomass recalcitrance and achieve efficient fractionation of biomass components. Cui et al. employed a synthesized deep eutectic solvent of choline chloride/lactic acid combined with ethanol for the pretreatment of Broussonetia papyrifera, which efficiently separated cellulose and lignin. When the deep eutectic solvent and ethanol were mixed at a ratio of 1:1, and the pretreatment conditions were set at 160°C for 1 h, the majority of hemicellulose and lignin in the residue was removed, resulting in the highest cellulose enzymatic hydrolysis efficiency of up to 46.25%. Furthermore, the study revealed that with an increase in pretreatment temperature, the content of phenolic hydroxyl groups in the separated lignin also increased, enhancing the antioxidant capacity of lignin.

## New catalytic biomass valorization strategies

The depolymerization of lignin to produce fuels and chemicals is crucial for converting lignocellulosic biomass into second-generation biofuels, promoting value-added utilization of lignin. Shao et al. employed formic acid as an *in-situ* hydrogen donor, and synergistically catalyzed the depolymerization of lignin using a noble metal catalyst Ru/C in combination with Lewis acid zinc chloride. Under the optimal process conditions, the bio-oil yield reached 91.1 wt%, with monomer yield accounting for 13.4 wt%. The presence of Lewis acid promoted the formation of monomeric guaiacyl compounds and 2,3-dihydrobenzofurans during the reaction.

Compared to lignin, carbohydrates have a simpler structure and can be industrially prepared as chemicals, especially hemicellulose. Currently, cellulose and hemicellulose can be efficiently and selectively converted to produce a range of platform chemicals, which can increase the profitability of biomass refining. Hemicellulose-derived furfural is a sustainable alternative to petrochemical intermediates in bulk chemical and fuel production. However, existing methods for converting xylose or hemicellulose in single/dual-phase systems involve non-selective sugar separation or lignin condensation, limiting the value-added utilization of lignocellulosic biomass. Previously, Huang et al. achieved efficient biomass separation using an aldehyde protection system, obtaining diformylxylose. This aldehyde-protected xylose derivative can efficiently produce furfural in a dual-phase system. In a water-methyl isobutyl ketone biphase system, diformylxylose can be converted to more than 76 mol% furfural at higher temperatures and shorter reaction times, which is more than twice the conversion efficiency of free xylose without aldehyde protection. Moreover, due to the advantages of separating highly active lignin using the formaldehyde-stabilized fractionation method, this combined approach not only improves the conversion efficiency from hemicellulose to furfural but also promotes the comprehensive utilization of the three main biopolymers in lignocellulose.

He et al. developed an efficient cascade reaction combining chemical catalysis and enzymatic biocatalysis to convert hemicellulose in lignocellulose to furfurylamine. Firstly, solid acid catalysis was used in a deep eutectic solvent composed of ethylenediamine hydrochloride and glycerol to convert hemicellulose to furfural efficiently. Subsequently, in the presence of NH_4_Cl (as the amine donor), *Escherichia coli* CCZU-XLS160 cells effectively aminated the formed furfural to furfurylamine with a yield exceeding 99%.

## Applications of lignocellulosic materials and derivatives in related fields

Currently, efficient separation of cellulose, hemicellulose, and lignin from lignocellulosic biomass can be achieved through specific isolation methods, promoting high-value utilization of individual components. Food safety is of paramount importance, making the production of food-safe detection materials from biobased materials highly attractive. Qin et al. successfully prepared starch films with pH-responsive and sensitive current responses by introducing innovative fractionation techniques, advanced characterization methods, and metabolic engineering strategies for carboxymethyl cellulose into starch. These films hold potential applications in rapid and real-time detection of liquid safety. Additionally, Xiang et al. utilized natural macromolecule xylan as a reducing and stabilizing agent to successfully synthesize stable bimetallic gold-silver nanoparticles, which were immobilized on paper surfaces to create paper-based surface-enhanced Raman scattering materials. This material effectively detected trace amounts of pesticides, showcasing the significant potential for rapid food safety monitoring.

## Conclusion

This Research Topic represents a significant advancement in lignocellulosic biomass valorization. The articles collectively emphasize the need to exploit genetic, biotechnological, and chemical approaches to fully understand the heterogeneous structure of lignocellulose. Researchers can efficiently convert lignocellulose into high-value chemical products and functional materials through thermochemical and biochemical conversion by employing innovative fractionation techniques, advanced characterization methods, and metabolic engineering strategies. The successful implementation of these approaches holds great promise for achieving large-scale, cost-effective production of biofuels and biomaterials, contributing to a more sustainable and renewable future.
